# The role and mechanism of macrophage-to-myofibroblast transition in fibrotic diseases

**DOI:** 10.3389/fimmu.2026.1919029

**Published:** 2026-10-05

**Authors:** Hua Guo, Xiao Xiao Tang

**Affiliations:** 1 State Key Laboratory of Respiratory Disease, National Clinical Research Center for Respiratory Disease, National Center for Respiratory Medicine, Guangzhou Institute of Respiratory Health, The First Affiliated Hospital of Guangzhou Medical University, Guangzhou, China; 2 Guangzhou National Laboratory, Bio-island, Guangzhou, China

**Keywords:** cellular origin, fibrotic diseases, mechanisms, MMT, therapeutic implications

## Abstract

Fibrosis is a pathological process primarily characterized by excessive deposition of extracellular matrix (ECM), which leads to disruption of tissue architecture and progressive loss of organ function, serving as a key pathological basis for various chronic diseases. Recent studies have revealed that, in addition to conventional sources, macrophages can directly differentiate into ECM-producing myofibroblasts through a process termed macrophage-to-myofibroblast transition (MMT), thereby actively participating in fibrogenesis. The concept of MMT was initially proposed in the context of renal fibrosis and has since been validated in multiple organs, including the kidney, heart, lung, liver, and retina. This review aims to summarize the role of MMT in fibrotic diseases and to elucidate the mechanism underlying MMT and fibrosis progression. Unlike prior reviews that predominantly focus on MMT within individual organs or separately delineate their molecular pathways, this review adopts a cross-organ perspective to systematically identify conserved mechanisms, tissue-specific regulatory networks, and translational challenges of MMT across fibrotic diseases.

## Introduction

Fibrosis is a pathological process characterized by excessive deposition of extracellular matrix (ECM) components, leading to disruption of tissue architecture and progressive loss of organ function (1–3). Fibrosis can occur in virtually all organ systems; its sustained progression disrupts normal tissue architecture and impairs organ function, ultimately leading to organ failure. Statistically, approximately 45% of all deaths worldwide are associated with fibrosis-related diseases. Common conditions linked to fibrosis include metabolic dysfunction-associated steatotic liver disease (MASLD), chronic kidney disease (CKD), idiopathic pulmonary fibrosis (IPF), and cardiovascular diseases, affecting nearly every organ system in the human body. With the annual incidence of major fibrotic diseases approaching 5%, fibrosis has emerged as a significant global public health concern, estimated to affect approximately 25% of the world’s population (4). From a pathophysiological perspective, fibrosis is closely intertwined with the wound healing process; both are regulated by similar cell populations and share multiple molecular mechanisms (5).

In recent years, substantial progress has been made in investigating key molecular pathways involved in ameliorating fibrotic organ dysfunction (6). Nevertheless, most current studies remain grounded in traditional mechanisms of fibrosis development, and consequently, the overall efficacy of existing therapeutic strategies remains limited (7). A variety of inflammatory cytokines, growth factors, and signaling pathways have been shown to promote fibrogenesis, indicating that interventions targeting these key mediators may represent promising anti-fibrotic therapeutic strategies (8). To date, the U.S. Food and Drug Administration (FDA) has approved only a few agents specifically for fibrotic diseases, including pirfenidone and nintedanib for idiopathic pulmonary fibrosis, as well as resmetirom and semaglutide for metabolic dysfunction-associated steatohepatitis (MASH) (9, 10). However, these drugs primarily function by slowing disease progression and remain limited in their ability to effectively reverse established pulmonary or hepatic fibrosis. Therefore, further elucidation of the mechanisms underlying fibrosis development and progression, as well as the exploration of novel therapeutic targets, is of great significance for developing more effective anti-fibrotic strategies.

Fibroblasts serve as core effector cells in the fibrotic process. Their activation not only drives ECM synthesis and deposition and facilitates fibrotic loci formation through differentiation into myofibroblasts but also plays a critical role in the cascade regulation of inflammatory signals. Consequently, targeting myofibroblast generation has become a key strategy in anti-fibrotic therapy: by inhibiting collagen synthesis and alleviating tissue remodeling, this approach ultimately aims to preserve and restore organ function. Studies have revealed marked heterogeneity in the origins of myofibroblasts within the fibrotic microenvironment. In addition to direct activation of resident fibroblasts, epithelial cells, endothelial cells, and pericytes can transdifferentiate into pro-fibrotic myofibroblasts through pathways including epithelial-to-mesenchymal transition (EMT), endothelial-to-mesenchymal transition (EndoMT), and pericyte-to-myofibroblast transition (PMT), respectively (11–14). Of particular interest, macrophages also possess the potential to differentiate into myofibroblasts—a process defined as macrophage-to-myofibroblast transition (MMT). MMT constitutes a distinct cellular mechanism that functionally couples chronic inflammation to fibroblast-mediated extracellular matrix deposition, offering novel mechanistic insights into the translation of immune dysregulation into sustained and irreversible fibrotic remodeling. Current evidence has confirmed MMT as a key mechanism mediating tissue remodeling in the kidney, heart, and retina (15–17); nevertheless, its deeper role in the pathological progression of fibrosis remains to be systematically elucidated. In the following sections, this review focuses on the research advances regarding MMT in fibrotic diseases.

## MMT

The concept of MMT was formally introduced in 2014 by Stephen Nikolic-Paterson and colleagues during their investigation of renal fibrosis. MMT describes the process by which bone marrow-derived macrophages differentiate into myofibroblasts following organ injury, thereby promoting the onset and progression of fibrosis (18). The proposal of MMT was not an abrupt development but rather the result of long-term observational accumulation. As early as 1972, researchers observed spindle-shaped cells with a morphology intermediate between macrophages and fibroblasts around peritoneal implants (19). In 1994, Richard Bucala and colleagues identified a population of bone marrow-derived circulating cells at wound sites that exhibited a fibroblast-like morphology and expressed monocyte antigens, which they termed “fibrocytes” (20, 21). These early findings laid the foundation for subsequent understanding of macrophage plasticity and its role in fibrosis. Although the precise mechanism between fibrocytes and MMT remains to be elucidated, subsequent studies employing marker detection and genetic analyses have confirmed that during tissue fibrosis, a subset of bone marrow-derived monocytes, upon recruitment to the injury site, can differentiate into macrophages and further transdifferentiate into myofibroblasts (22). In recent years, multiple studies have demonstrated the presence of MMT in various organ systems, suggesting that MMT may be widely involved in the pathogenesis of diverse fibrotic diseases, thereby representing a potential therapeutic target for intervention in fibrosis progression (23).

Currently, the primary methods and indicators for evaluating MMT include the following: co-localization analysis of markers, immunofluorescence staining or flow cytometry to detect co-expression of monocyte/macrophage markers (e.g., CD68, F4/80) and fibroblast/myofibroblast markers (e.g., alpha-smooth muscle actin [α-SMA], type I collagen, fibroblast-specific protein 1 [FSP1], fibroblast activation protein [FAP]) (23); *in vitro* induction models, employing transforming growth factor-beta 1 (TGF-β1) or other stimulating factors to induce macrophage cell lines (e.g., RAW264.7, THP-1, or MH-S) or primary macrophages, followed by assessment of morphological changes and expression levels of relevant genes and proteins (24, 25); lineage tracing and transcriptomic analysis, using single-cell RNA sequencing or lineage-tracing mouse models (e.g., Cx3cr1Cre; Rosa26^Td^ mice) to track macrophage fate and demonstrate their transdifferentiation into myofibroblasts within the fibrotic microenvironment (26). Furthermore, mechanistic studies often employ inhibitors or pharmacological interventions targeting specific signaling pathways to elucidate the regulatory roles of key molecules in the MMT process (23). Collectively, multiple signaling pathways, with the TGF-β1/Smad3 axis at the core, orchestrate the transdifferentiation of macrophages into myofibroblasts during MMT (**Figure 1**).

**Figure 1:**
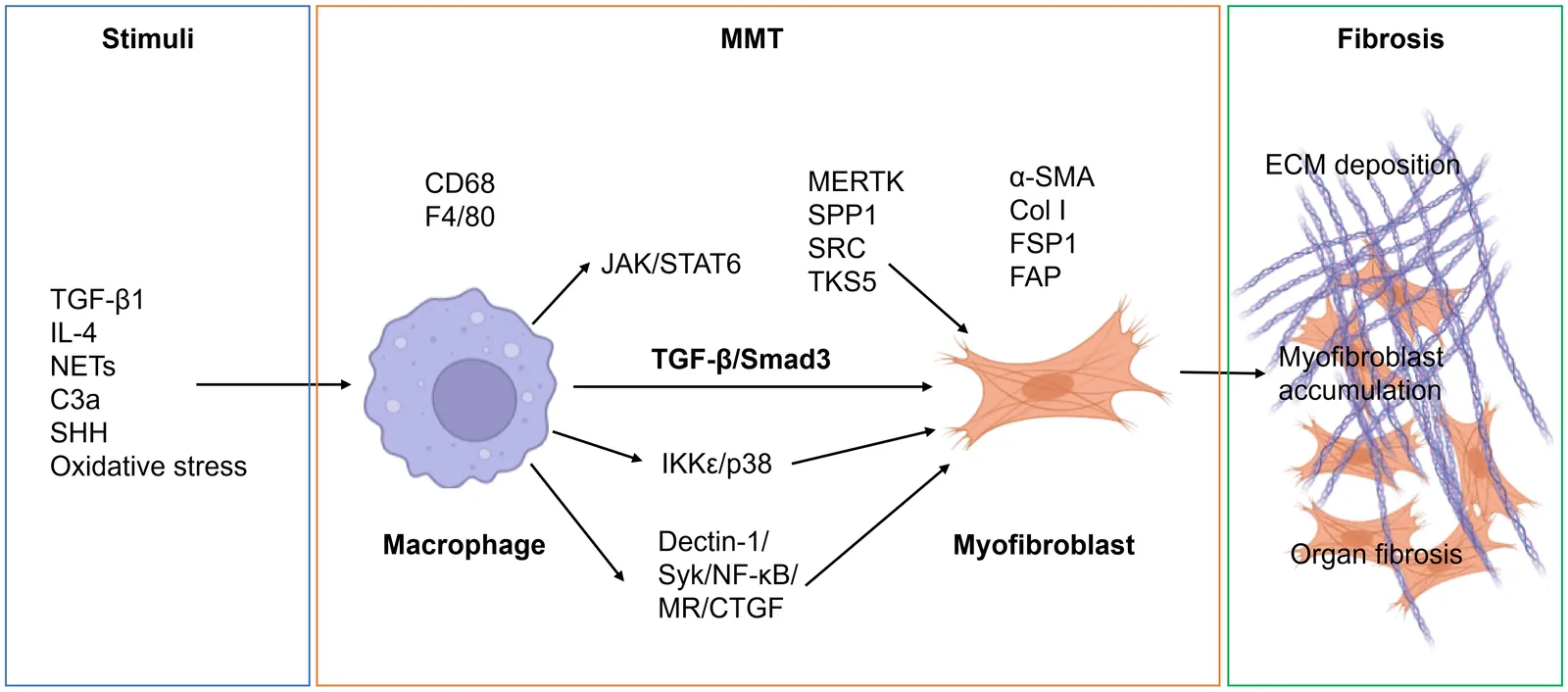
Molecular mechanisms of MMT. Multiple profibrotic stimuli (e.g., TGF-β1, interleukin-4 [IL-4]) converge on macrophages, activating key signaling pathways such as TGF-β1/Smad3, JAK/STAT6, MERTK/SPP1, and IKKϵ/p38. These cascades drive the transdifferentiation of CD68^+^CD206^+^ M2-like macrophages into myofibroblasts, ultimately promoting excessive ECM deposition and organ fibrosis. Positive and negative regulators of MMT are indicated.

Taken together, current evidence suggests that TGF-β1/Smad3 functions as the central signaling hub regulating MMT, whereas other signaling pathways mainly modulate macrophage activation, interact with the TGF-β1/Smad3 cascade, or act in an organ-specific manner. This integrated regulatory network provides multiple potential therapeutic targets for preventing fibrotic diseases.

## MMT in fibrotic diseases

### Kidney fibrosis

#### Evidence and cellular origin

The essence of renal fibrosis lies in excessive deposition of ECM, a pathological process that progressively replaces the normal renal tissue architecture and ultimately leads to kidney failure (27). Within the fibrotic process, myofibroblasts serve as the primary source of ECM, and their cellular origin has remained a focal point of investigation. In addition to resident fibroblasts, epithelial cells, and pericytes, accumulating evidence indicates that bone marrow-derived macrophages can differentiate into collagen-producing myofibroblasts through a process termed MMT, thereby playing a critical role in the progression of renal fibrosis (28, 29). In animal models of CKD, such as unilateral ureteral obstruction (UUO), lineage-tracing studies have revealed that a considerable proportion of α-SMA^+^ myofibroblasts originate from myeloid cells (30). The cells undergoing MMT exhibit a distinct phenotype, co-expressing the macrophage marker CD68 and the myofibroblast marker α-SMA, thereby forming transitional-state α-SMA^+^CD68^+^ cells. Notably, these cells also typically show high expression of CD206, a marker of M2-polarized macrophages, supporting the view that macrophages undergoing MMT predominantly display an M2 phenotype. This suggests that upon reaching the site of injury, circulating myeloid cells first polarize into M2 macrophages and subsequently transdifferentiate into pro-fibrotic myofibroblasts via MMT (30, 31).

#### Molecular mechanisms and therapeutic implications

At the molecular level, TGF-β1 and its downstream Smad3 signaling pathway are widely recognized as the central hub regulating MMT. TGF-β1 not only serves as a major inducer of M2 macrophage polarization but also drives the initiation and execution of MMT (32, 33). Studies have confirmed that conditional knockout of TGF-β receptor or Smad3 significantly reduces myofibroblast accumulation and attenuates fibrosis (34, 35). Furthermore, Src kinase, as a target gene of Smad3, plays a pivotal role in mediating the TGF-β1/Smad3 signaling and promoting MMT; pharmacological inhibition of Src blocks MMT and reduces ECM deposition (36–38). Using INK-ATTAC senescence-tracer mouse models, researchers have found that the expression of p16INK-4a and MMT markers is markedly upregulated in the kidney of renal artery stenosis (RAS) mice, whereas administration of a senolytic agent AP20187 alleviates renal fibrosis. Mechanistically, senescent renal tubular epithelial cells highly express IFITM3, which upregulates ITGB3 in macrophages via a paracrine mechanism, thereby activating the TGF-β/Smad3 pathway to drive MMT, while silencing IFITM3 or ITGB3 abrogates this effect (15). TGF-β1/Smad3 signaling is well-established as the core regulatory axis governing MMT progression. Nevertheless, a growing body of evidence indicates that MMT is orchestrated by a highly interconnected signaling network, rather than an isolated linear pathway. Other signaling pathways may act upstream of TGF-β1/Smad3, modulate Smad3 activation, or cooperate with this core cascade to regulate macrophage phenotypic transition across distinct tissue microenvironments. Among these regulatory pathways, JAK/STAT signaling represents an important modulatory axis that cooperates with the TGF-β1/Smad3 pathway to drive macrophage polarization and amplify downstream profibrotic responses. The JAK/STAT6 pathway (particularly IL-4-mediated STAT6 activation) has also been shown to participate in M2 macrophage polarization and subsequent MMT, and targeting this pathway demonstrates anti-fibrotic potential (39). Other factors, such as Pou4f1 (a transcriptional target of Smad3) (40), GSDMD-mediated neutrophil extracellular traps (NETs) (41), and the STING/TBK1 axis (42), have been reported to influence MMT by modulating or interacting with TGF-β1 signaling. Moreover, recent evidence indicates that the pattern recognition receptor Dectin-1 is upregulated in macrophages within fibrotic kidneys of CKD patients, and its expression level correlates positively with the degree of fibrosis and renal functional impairment. Genetic knockout or macrophage-specific deletion of Dectin-1 ameliorates UUO- or ischemia-reperfusion-induced renal fibrosis in mice. Mechanistically, Dectin-1 not only promotes macrophage infiltration via the Syk/NF-κB/CCL2-CCR2 axis but also activates the TGF-β/Smad signaling to drive MMT. Additionally, pharmacological inhibition of Dectin-1 using Laminarin alleviates renal fibrosis (43).

In summary, MMT serves as a critical bridge linking inflammation and fibrosis and has emerged as a frontier in renal fibrosis research. Targeting the MMT origin of myofibroblasts, in particular by modulating key signaling pathways such as TGF-β1/Smad3 and JAK/STAT6, provides a fundamental theoretical basis and promising interventional targets for the development of novel anti-fibrotic strategies in the treatment of CKD.

## Pulmonary fibrosis

### Evidence and cellular origin

Pulmonary fibrosis is pathologically characterized by excessive ECM accumulation, leading to progressive decline in lung function. Studies have demonstrated that MMT plays an important role in fibrotic tissue remodeling by increasing the population of myofibroblasts (8). Single-cell sequencing and fluorescence *in situ* hybridization have revealed co-expression of monocyte/macrophage and myofibroblast markers in fibrotic lung tissue. Notably, approximately 35% of myofibroblasts express M2-type marker CD206, suggesting that M2 polarization may represent a prerequisite stage for MMT (23, 44).

### Molecular mechanisms and therapeutic implications

Mechanistically, the TGF-β1-Smad2/3 signaling axis serves as the core pathway driving MMT, and macrophage-specific Smad3 silencing abrogates this process and inhibits disease progression (45, 46). Furthermore, Cell chemokine receptor 8 (CCR8) promotes MMT-mediated fibroblast formation by enhancing autophagy (47). In specific pathological contexts, such as around tuberculous granulomas, MMT is sequentially regulated by STAT1/STAT3 signaling and the inflammatory microenvironment (48). Recent interventional studies have shown that activating PGC-1α alleviates mitochondrial dysfunction and reduces mtDNA migrasome release, thereby suppressing MMT and ameliorating sepsis-associated pulmonary fibrosis (49). Moreover, penicillin has been found to inhibit bleomycin (BLM)-induced pulmonary fibrosis and MMT by targeting Frem1. Mechanistic investigations identified Frem1 as a key regulator that activates the MyD88/NF-κB pathway, thereby initiating a positive feedback loop of interleukin-1 beta (IL-1β) that drives fibrotic progression. Using genetic knockout approaches, the study delineated the essential role of Frem1 in MMT and demonstrated that penicillin exerts its anti-fibrotic effects primarily by interfering with the Frem1/NF-κB/IL-1β axis (50). In parallel, aberrant activation of erythropoietin receptor (EPOR) signaling in macrophages has emerged as a key mechanism driving MMT in pulmonary fibrosis. Integrative single-cell transcriptomic and gene-modified animal model analyses indicate that EPOR expression levels positively correlate with the extent of MMT and collagen deposition. Mechanistically, EPOR potentiates the TGF-β1/Smad3 signaling through activating the JAK2/STAT3 pathway and induces M2 polarization of macrophages, thereby creating a microenvironment conducive to fibrotic progression. Given the dual role of EPOR in regulating macrophage phenotypic switching and fibroblast activation, targeted therapy against the EPOR/JAK2/Smad3 axis may represent a novel strategy to reverse the pathological progression of pulmonary fibrosis (51). Studies have further identified a close association between MERTK and MMT. The MERTK signaling axis plays a key role in driving MMT by activating the SPP1/SRC/TKS5 cascade. Both *in vivo* and *in vitro* experiments have confirmed that targeted inhibition of key nodes in this axis (e.g., MERTK knockout or TKS5 knockdown) significantly improves lung function and reduces lung fibrosis. Thus, interrupting the MERTK-initiated signaling cascade not only represents an strategy to inhibit MMT but also offers novel insights for the treatment of pulmonary fibrosis (52, 53). Although the mechanisms underlying MMT in pulmonary fibrosis have garnered increasing attention, research in this area remains at an exploratory stage.

## Cardiac fibrosis

### Evidence and cellular origin

Cardiovascular disease remains one of the leading causes of death worldwide. As terminally differentiated cells, cardiomyocytes possess limited proliferative and self-renewal capacity; consequently, damaged myocardial tissue is primarily repaired through fibrotic scar formation (54). However, excessive fibrosis reduces ventricular compliance and ultimately precipitates heart failure. MMT plays a significant role in this pathological process. Activation of the ALKBH5/interleukin-11 (IL-11)/IL11RA1 signaling axis has been shown to induce MMT and promote phenotypic conversion of cardiac macrophages, thereby mediating cardiac fibrosis and dysfunction in a mouse model of hypertension. Notably, a lipid nanoparticle (LNP)-based siRNA strategy targeting this pathway holds promise as a potential therapeutic approach for cardiac fibrosis (26). *In vitro* studies have demonstrated that human CD14^+^ monocytes, following phorbol 12-myristate 13-acetate (PMA)-induced differentiation into macrophages, gradually acquire a spindle-shaped morphology during prolonged culture and express myofibroblast markers such as S100A4, α-SMA, col1α1, and P4H in a time-dependent manner. Lineage-tracing studies further revealed an increased number of cells co-expressing YFP and α-SMA in the hearts of LysM^Cre/+^ROSA26^EYFP/+^ transgenic mice six weeks after myocardial ischemia-reperfusion (MIR), confirming the involvement of MMT in cardiac fibrosis (55). An integrative omics-based study across four cardiac conditions—dilated cardiomyopathy, ischemic cardiomyopathy, myocardial infarction, and heart failure—identified MMT as a key mechanism promoting myocardial fibrosis. In these conditions, profibrotic macrophages exhibited M1 polarization and secreted fibrosis-related molecules such as FN1, SPP1, and THBS1, contributing to extracellular matrix remodeling. Moreover, in heart failure patients who responded positively to left ventricular assist device therapy, MMT-related marker expression was significantly reduced, suggesting the reversibility of MMT. Furthermore, multiple driver genes (e.g., RPS2, RPL3, RPS9, EEF1A1) and transcription factors (e.g., FOS, ATF3, EGR1) may participate in MMT by regulating RNA metabolism and transcriptional processes, underscoring MMT as a critical cellular transition in macrophage-mediated myocardial fibrosis with significant pathological and therapeutic implications (56).

### Molecular mechanisms and therapeutic implications

Mechanistically, another study employing single-cell sequencing and phosphoprotein array technologies revealed that the IKKϵ-p38 axis plays an important role in resolving inflammation and promoting cardiac repair following myocardial infarction. Loss of IKKϵ leads to reduced p38 phosphorylation, accelerating MMT in a specific macrophage subpopulation, thereby exacerbating inflammatory responses and cardiac fibrosis, ultimately impairing cardiac function. Conversely, treatment with 5-azacytidine (5-Aza) restores phosphorylated p38 levels and inhibits MMT, suggesting that IKKϵ-p38-mediated MMT represents a promising therapeutic target in ischemic heart disease (16). In patients with CKD, cardiovascular complications are the leading cause of mortality; however, the underlying mechanisms of CKD-associated cardiac pathology remain incompletely understood. In a UUO-induced model of kidney injury, the heart exhibits pronounced fibrosis and MMT. Further mechanistic studies have demonstrated that aldosterone induces MMT and promotes secretion of connective tissue growth factor (CTGF) through activation of the mineralocorticoid receptor (MR), with CTGF in turn positively reinforcing MMT. Inhibition of the MR/CTGF pathway using eplerenone or CTGF-blocking agents reverses these effects, highlighting that targeting MMT and its regulatory axis is a potential strategy for intervening in CKD-related cardiac fibrosis (57). Despite the accumulating evidence underscoring the importance of MMT in cardiac fibrosis, research in this area remains relatively limited, and further investigation into the underlying mechanisms and clinical translation is warranted.

## Hepatic fibrosis

Hepatic fibrosis is a pathophysiological response of the organism to chronic injuries such as viral infection, drug toxicity, alcoholic and non-alcoholic fatty liver disease. Immunofluorescence analyses of clinical hepatic fibrosis specimens and various chronic liver injury models—including carbon tetrachloride-induced liver injury, non-alcoholic fatty liver disease models induced by high-fat diet or methionine-choline-deficient diet, and ethanol-induced alcoholic fatty liver disease—have revealed the widespread presence of CD68^+^α-SMA^+^ co-expressing MMT cells. These cells predominantly exhibit an M2-polarized phenotype, confirming that MMT plays a significant role in the progression of chronic liver injury and fibrosis (58). Furthermore, caspase-4-dependent neutrophil activation and GSDMD-mediated processes promote the formation of NETs. The extracellular DNA released by NETs further facilitates MMT driven by the TGF-β1/Smad3 signaling pathway. Inhibition of GSDMD suppresses cirrhosis-associated portal vein thrombosis and prevents the associated fibrogenesis (59).

## Retinal fibrosis

Research has also shown that macrophage-derived MMP12 is upregulated in subretinal fibrosis and mediates TGF-β-induced MMT, while inhibition of MMP12 alleviates fibrosis (60). Moreover, MMT may promote the formation of myofibroblasts in the epiretinal membrane, and this transformation predominantly involves macrophages with an M2 phenotype. Concurrently, FAP-α, a vitreous biomarker, may originate from the transition of M2 macrophages into myofibroblasts (17). In macular fibrosis associated with neovascular age-related macular degeneration, complement C3a can also induce MMT in macrophages, a process that can be blocked by a C3aR antagonist (61). A recent study found that in subretinal fibrosis (SF), VEGF stimulation upregulates RASA1 expression in human umbilical vein endothelial cells (HUVECs), thereby enhancing SHH secretion. The secreted SHH further activates the SHH-SMO/PTCH1-GLI1 axis in macrophages, inducing MMT and accelerating fibrogenesis (62). Another study confirmed that NR4A1 acts as an endogenous inhibitor of MMT and can reduce ECM deposition in SF. The therapeutic effect of its agonist, Csn-B, is primarily achieved by inhibiting the AKT/NR4A1 phosphorylation axis, thereby blocking the phenotypic transition of macrophages into myofibroblasts and mitigating SF progression (63). Furthermore, a novel regulatory axis, circ_0001103/miR-7240-5p/SLC9A1, has been revealed to play a key role in MMT-mediated subretinal fibrosis. Inhibition of circ_0001103 not only alleviates fibrosis by remodeling macrophage phenotypes but also curbs pathological leakage in choroidal neovascularization (24).

## Fibrosis in other organs

Transcriptomic analyses reveal monocytes derived from patients with pancreatic ductal adenocarcinoma already display pronounced fibrotic characteristics. Under oxidative stress, reactive oxygen species can induce and promote MMT progression by mediating p53 stabilization or activating the p38-MAPK pathway (64). These findings provide a molecular basis and highlight not only the ubiquity of MMT but also its potential as a therapeutic target. MMT has now been documented across multiple organ systems, including the kidney, lung, heart, liver, and retina, underscoring its conserved role in fibrotic pathogenesis (**Figure 2**). The key characteristics, signaling pathways, and potential therapeutic interventions of MMT in different organs are summarized in **Table 1**. While MMT shares conserved core features across distinct fibrotic organs, accumulating evidence indicates that its regulatory programs are highly contingent on tissue-specific microenvironments. We therefore summarize both the universal and organ-unique mechanisms governing MMT across different fibrotic disease contexts (**Table 2**).

**Figure 2:**
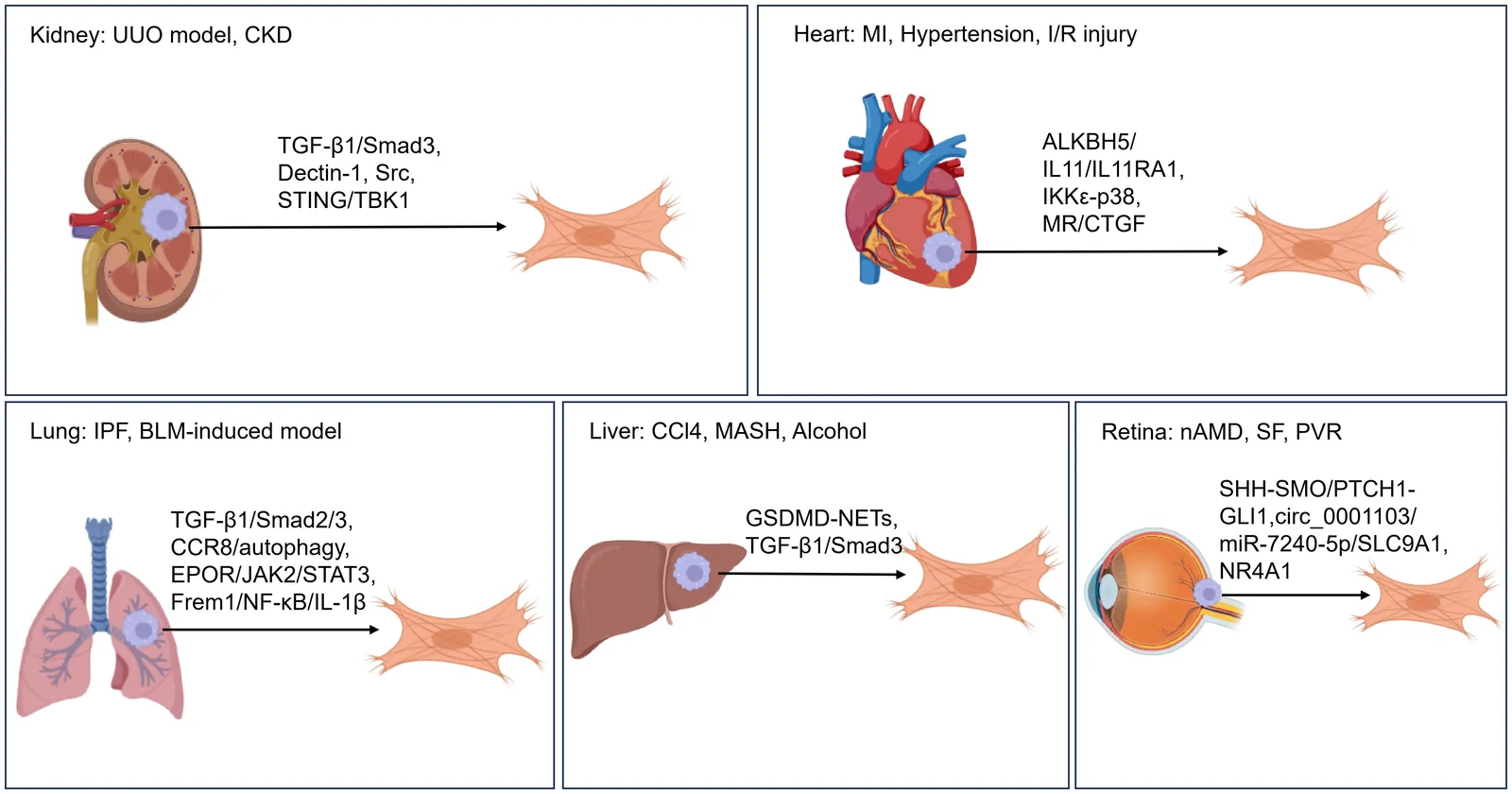
Organ-specific involvement of MMT. MMT has been identified as a conserved profibrotic mechanism in the kidney (UUO, CKD), lung (IPF, BLM-induced), heart (MI, hypertension), liver (CCl4, MASH), and retina (nAMD, SF).

**Table 1:** Characteristics and mechanisms of MMT in multi-organ fibrosis.

Organ system	Pathological context	Key signaling pathways & molecular mechanisms	Therapeutic strategies/potential interventions	Reference numbers
Kidney	CKD, UUO model	Central Hub: TGF-β1/Smad3 axis; Regulators: Src kinase, JAK/STAT6, Dectin-1/Syk/NF-κB, Pou4f1	Src inhibitors, AP20187 (Senolytic), Laminarin (Dectin-1 inhibitor), Eplerenone (MR antagonist)	(15, 30–33, 36–39, 43)
Lung	IPF, BLM-induced fibrosis	Core Axis: TGF-β1/Smad2/3; Drivers: MERTK/SPP1/SRC/TKS5 cascade, EPOR/JAK2/STAT3, Frem1/NF-κB/IL-1β axis	Penicillin (targeting Frem1), PGC-1α activation, MERTK knockout or TKS5 knockdown	(8, 23, 44–46, 49–51, 53)
Heart	MI, Hypertension, Ischemia-Reperfusion injury	Signaling Axis: ALKBH5/IL-11/IL11RA1; Regulatory: IKKϵ-p38 axis, MR/CTGF pathway	LNP-siRNA targeting ALKBH5, 5-Aza, Eplerenone or CTGF-blocking agents	(16, 26, 55–57)
Liver	Cirrhosis, MASLD/MASH, Alcoholic Liver Disease	Inflammatory Interaction: GSDMD-mediated NETs, TGF-β1/Smad3 signaling	GSDMD inhibition	(58, 59)
Retina/Eye	SF, Macular Fibrosis, Vitreoretinal Disorders	Regulatory Axis: SHH-SMO/PTCH1-GLI1, circ_0001103/miR-7240-5p/SLC9A1, AKT/NR4A1 axis	C3aR antagonist, Csn-B (NR4A1 agonist), circ_0001103 inhibition, MMP12 inhibition	(17, 24, 61–64)

This table summarizes the multi-organ involvement of MMT. In each organ system—including the kidney, lung, heart, liver, and retina—the transition is characterized by the emergence of transitional cells co-expressing macrophage markers (e.g., CD68, CD206) and myofibroblast markers (e.g., α-SMA). The TGF-β1/Smad3 signaling axis serves as the ubiquitous central hub driving this process, while organ-specific regulators—such as Dectin-1 in the kidney, MERTK in the lung, and ALKBH5 in the heart —fine-tune the fibrotic response.

**Table 2:** Universal and organ-unique mechanisms governing MMT across fibrotic diseases.

Organ	Evidence	Universal mechanisms	Organ-unique mechanisms	Level of evidence
Kidney	Lineage tracing; CD68^+^α-SMA^+^ cells	TGF-β1/Smad3	Dectin-1, STING/TBK1	Strong
Lung	scRNA-seq, animal models	TGF-β signaling	MERTK/SPP1/SRC/TKS5, EPOR	Moderate-Strong
Heart	Lineage tracing, omics analysis	macrophage plasticity	IKKϵ-p38, ALKBH5/IL-11	Moderate
Liver	Human samples; fibrosis models	TGF-β signaling	NETs/GSDMD-mediated regulation	Moderate
Retina	Human samples, models	TGF-β-driven MMT	SHH, NR4A1, circRNA axis	Preliminary-Moderate

## Summary and perspective

The pathological core of fibrotic diseases lies in the persistent activation of myofibroblasts and the excessive deposition of ECM. As a critical bridge linking chronic inflammation to fibrosis, MMT has been recognized as an important contributor to the myofibroblast pool across multi-organ fibrosis. Since the formal designation of MMT in 2014, the academic understanding of myofibroblast heterogeneity has expanded from traditional concepts of *in situ* fibroblast activation and EMT to encompass the plasticity regulation of bone marrow-derived immune cells. However, whether MMT represents a quantitatively dominant source of myofibroblasts in human fibrotic diseases remains an open question, as its contribution may vary depending on disease stage, tissue context, and experimental systems. Numerous studies, employing lineage tracing, co-localization of biomarkers, and single-cell transcriptomic sequencing, have confirmed the presence of CD68^+^α-SMA^+^ transitional cells in various fibrotic models involving the kidney, heart, lung, and liver. These investigations have identified the TGF-β1/Smad3 signaling axis as the central driver of this transdifferentiation process, while alternative pathways such as JAK/STAT, MERTK/SPP1, and IKKϵ-p38, through crosstalk with the core pathway, finely regulate the irreversible transition of macrophages toward a pro-fibrotic phenotype. Notably, macrophages undergoing MMT predominantly exhibit an M2-polarized background, a phenomenon that not only tightly couples the immunosuppressive microenvironment with tissue remodeling mechanisms but also suggests that targeting MMT may serve as a dual-effect strategy to simultaneously intervene in persistent inflammation and matrix deposition.

Although interventions targeting MMT have demonstrated significant potential for alleviating fibrosis in preclinical models, current research still faces numerous challenges. First, static detection of CD68^+^α-SMA^+^ co-expression is insufficient to fully delineate the continuous lineage dynamics of cellular states during MMT, necessitating the introduction of spatial multi-omics and high-resolution intravital imaging to elucidate the critical molecular events and spatial niche characteristics of this transition. Second, the temporal switch between the early protective functions of macrophages in debris clearance and their later pro-fibrotic remodeling remains poorly defined, with a lack of precise biomarkers and specific regulatory tools. Furthermore, the initiation mechanisms, spatiotemporal dynamics, and the impact of macrophage heterogeneity on the transdifferentiation trajectory of MMT require further elucidation. Future breakthroughs should focus on the epigenetic remodeling and metabolic checkpoint mechanisms driving trans-lineage transdifferentiation, integrating single-cell multi-omics, lineage tracing, and gene editing technologies to deeply dissect the metabolic regulatory networks and intercellular crosstalk. On this basis, developing novel targeted delivery systems or engineered cell therapies capable of temporal cellular recognition, and screening highly specific interventional agents, will be crucial to block the supply of MMT-derived myofibroblasts while preserving the essential immune surveillance and repair-initiating functions of macrophages. From a broader perspective, MMT research not only deepens our understanding of the paradox of irreversible fibrosis but also provides a novel theoretical foundation and a highly translatable clinical strategy for shifting anti-fibrotic therapies from passively delaying matrix accumulation to actively intervening in cell fate determination.
